# β-Hexachlorocyclohexane: A Small Molecule with a Big Impact on Human Cellular Biochemistry

**DOI:** 10.3390/biomedicines8110505

**Published:** 2020-11-16

**Authors:** Elisabetta Rubini, Giuliano Paglia, David Cannella, Alberto Macone, Antonella Di Sotto, Marco Gullì, Fabio Altieri, Margherita Eufemi

**Affiliations:** 1 Department of Biochemical Science “A. Rossi Fanelli”, Faculty of Pharmacy and Medicine, Sapienza University of Rome, P.le Aldo Moro 5, 00185 Rome, Italy; elisabetta.rubini@uniroma1.it (E.R.); giuliano.paglia@uniroma1.it (G.P.); alberto.macone@uniroma1.it (A.M.); fabio.altieri@uniroma1.it (F.A.); 2 PhotoBioCatalysis Unit–Bio-Cat, Interfaculty School of Bioengineers, Université libre de Bruxelles, CP245, Bd du Triomphe, 1050 Brussels, Belgium; david.cannella@ulb.ac.be; 3 Department of Physiology and Pharmacology “V. Erspamer”, Faculty of Pharmacy and Medicine, Sapienza University of Rome, P.le Aldo Moro 5, 00185 Rome, Italy; antonella.disotto@uniroma1.it (A.D.S.); marco.gulli@uniroma1.it (M.G.)

**Keywords:** β-hexachlorocyclohexane, organochlorine pesticides, endocrine disrupting chemical, aryl hydrocarbon receptor, oxidative stress, DNA damage, energy metabolism, cancer, environmental pollution

## Abstract

Organochlorine pesticides (OCPs) belong to a heterogeneous class of organic compounds blacklisted by the Stockholm Convention in 2009 due to their harmful impact on human health. Among OCPs, β-hexachlorocyclohexane (β-HCH) is one of the most widespread and, at the same time, poorly studied environmental contaminant. Due to its physicochemical properties, β-HCH is the most hazardous of all HCH isomers; therefore, clarifying the mechanisms underlying its molecular action could provide further elements to draw the biochemical profile of this OCP. For this purpose, LNCaP and HepG2 cell lines were used as models and were subjected to immunoblot, immunofluorescence, and RT-qPCR analysis to follow the expression and mRNA levels, together with the distribution, of key biomolecules involved in the intracellular responses to β-HCH. In parallel, variations in redox homeostasis and cellular bioenergetic profile were monitored to have a complete overview of β-HCH effects. Obtained results strongly support the hypothesis that β-HCH could be an endocrine disrupting chemical as well as an activator of AhR signaling, promoting the establishment of an oxidative stress condition and a cellular metabolic shift toward aerobic glycolysis. In this altered context, β-HCH can also induce DNA damage through H2AX phosphorylation, demonstrating its multifaceted mechanisms of action.

## 1. Introduction

Organochlorine pesticides (OCPs) represent 40% of total environmental pollutants and constitute a significant source of contamination all over the world. OCPs exhibit a related chemical structure characterized by aliphatic or aromatic chlorine-substituted rings and their hazardousness is mostly due to shared physicochemical properties, such as lipophilia and energetic stability, responsible for the persistence and high bioaccumulation potential of these molecules [1].

In recent years, growing attention has been paid to the hexachlorocyclohexane (HCH), a chlorinated cyclic saturated hydrocarbon listed as “POP” (persistent organic pollutant) by the Stockholm Convention and finally banned in 2009 [2].

The industrial synthesis of hexachlorocyclohexane through the benzene photochlorination gives a mixture of isomers (α,β,γ,δ,ε) that structurally differ in the axial and equatorial orientation of the chlorine atoms with respect to the cyclohexane carbon ring. Among these isomers, only γ-HCH has specific insecticidal properties and it is referred to as lindane [3].

The extensive use of lindane as a commercial pesticide during the last 70 years is a heavy burden for human beings as well as the environment: 8–12 tons of undesired derivatives were produced for each ton of lindane, leading to an overall storage of almost 7.2 million tons of HCH waste isomers mostly buried in uncontrolled dumps at many sites around the world [4].

Compared to other HCH isomers, β-HCH is more lipophilic and is the most physically and chemically stable due to the equatorial position of all the six chlorine atoms in the chair cyclohexane conformation; this stability is reflected in the environmental and biological persistence of this molecule [5].

Despite its worldwide distribution, knowledge of β-HCH effects on human health is controversial and limited to studies in workers employed in the use and production of this pesticide.

By typing “β-hexachlorocyclohexane” into PubMed search engine (link: https://pubmed.ncbi.nlm.nih.gov), the results-by-year timeline shows 450 publications spanning from 1975 to 2019; after adding the word “disease” to narrow the field and focus on the biological consequences of β-HCH contamination, the number of available papers is reduced to 63.

Conversely, literature outcomes for the HCH aromatic analogous hexachlorobenzene provide 2946 scientific papers, of which 242 are found including the keyword “disease” in the search criteria. Such a difference between the two compounds could be explained by the fact that β-HCH is considered a waste by-product without insecticidal activity.

Even so, β-HCH is potentially one of the main contributors to the so-called civilization diseases, which are pathological conditions (i.e., cancer, neurodegenerative diseases, metabolic disorders) mostly linked to exogenous factors rather than to an intrinsic impairment of human physiological processes.

But what are the molecular mechanisms underlying β-HCH toxicity? Taking into account the tight correlation between structure and function of biomolecules, it is conceivable to hypothesize that, as well as other similar substances, β-HCH may:

1. Act as an endocrine-disrupting chemical by interfering with hormone cascades;
2. interact with the Aryl Hydrocarbon Receptor (AhR), the xenobiotic sensor par excellence;
3. induce oxidative stress, consequently affecting energy homeostasis and metabolism; and
4. cause DNA damage.

Our research group is investigating, as of 2015, the molecular effects of β-HCH using different types of human cancer cell lines as an in vitro model. Our findings assessed the central role of the oncoprotein STAT3 [6] in mediating the intracellular responses to β-HCH, which can activate different receptors and their related signaling pathways in a cell line-specific manner [7].

Principles of functional biochemistry point out how the activation/inhibition of cellular processes is often dose-dependent and crucially determined by the structural affinity of a molecule for its bio-targets. However, in the case of a large excess, the ligand can interact with various off targets.

To clarify whether β-HCH at the exposure concentration value could be able to trigger the above-mentioned processes, the experimental molarity was extrapolated from an epidemiological study, published in 2016 from the Epidemiological Department of Lazio and still ongoing, which is biomonitoring β-HCH blood levels in a sample of 690 exposed patients living close the Valle del Sacco, an industrial area in the south of Rome [8].

## 2. Experimental Section

### 2.1. Cell Cultures

Human prostate cancer cell line LNCaP and hepatocellular carcinoma cell line HepG2 were purchased from American Type Culture Collection (ATCC).

Cells were grown to 80% confluence at 37 °C and 5% CO_2_ in the appropriate culture medium, RPMI 1640 (Sigma-Aldrich, Milano, Italy cat. R0883) or DMEM-LG (Sigma-Aldrich, Milano, Italy, cat. D5546), supplemented with 1% sodium pyruvate, 10% fetal bovine serum, 2 mM glutamine, 100 µg/mL streptomycin and 100 U/mL penicillin. Cells were treated with 10 µM β-HCH (Sigma-Aldrich, Milano, Italy, cat. 33376) and 30 nM Testosterone (Sigma-Aldrich, Milano, Italy, cat. 86500) at variable time-points depending on the experiment type. For the inhibition each cell line was pre-treated or not with 120 nM bicalutamide (Sigma Aldrich, Milano, Italy, cat. B9061), 150 nM CH223191 (Sigma Aldrich, Milano, Italy, cat. C8124) or 2 µM MG-132 (Sigma Aldrich, Milano, Italy, cat. M8699).

To follow the receptors nuclear translocation, cells were treated for 4 h with 10 µM β-HCH or 30 nM testosterone. In order to assess the AR agonism of β-HCH, cells were subjected to an overnight pre-treatment with 120 nM bicalutamide before 4 h of incubation with 10 µM β-HCH or 30 nM testosterone. To induce oxidative stress and DNA damage, 75 µM tert-butyl hydro peroxide was used for 1 h (Sigma-Aldrich, Milano, Italy, 458139). Instead, to demonstrate the activation of AhR, cells were incubated for 4 h with 10 µM β-HCH following a 2 h pre-treatment with 150 nM CH223191 or 2 µM MG-132. ROS production, GSH/GSSG ratio and lactate/pyruvate ratio were determined following 6 h treatment with 10 µM β-HCH. DNA damage was observed after a treatment of 4 h.

### 2.2. Protein Extraction and Immunoblotting

Protein extraction and immunoblotting analysis were performed essentially according to Rubini et al. [7]. Cells cultured on 6-well plates were scraped, harvested by centrifugation, and washed in PBS. Total protein extracts were obtained using a lysis buffer containing 2% SDS, 20 mM Tris-hydrochloride pH 7.4, 2 M urea, 10% glycerol added with 2 mM sodium orthovanadate, 10 mM DTT, and a protease inhibitors cocktail diluted 1:100 (Sigma-Aldrich, Milano, Italy). Nuclei were obtained from cell pellets using a hypotonic buffer (10 mM HEPES, pH 7.5), 10 mM KCl, 1.5 mM MgCl_2_, 0.5 mM DTT) added with 0.05% Triton-X, 2 mM sodium orthovanadate, and a protease inhibitors cocktail diluted 1:100 (Sigma-Aldrich, Milano, Italy). Thus, nuclei were harvested by centrifugation and washed in a hypotonic buffer, and nuclear protein extracts were obtained as described above for total protein extracts. Proteins were resolved by SDS-PAGE 10% TGX FastCastTM Acrylamide gel (BioRad, Segrate, Italy, cat. 161-0183) and transferred on PVDF membranes using Trans-Blot^®^ TurboTM Transfer System (BioRad, Segrate, Italy, cat. 170-4247). The membranes were blocked with 3% *w*/*v* non-fat dried milk or 0.2% *w*/*v* I-block (Thermo Fisher Scientific, T2015, Monza, Italy) in Tris-buffered saline containing 0.05% Tween-20 (TBS-T) and incubated with a specific primary antibody for 1 h. Subsequently, membranes were washed three times in TBS-T, and then incubated for an additional hour with appropriate alkaline phosphatase-conjugated secondary antibody (Sigma-Aldrich, Milano, Italy, cat. A3687-A3688, dilution 1:5000). The alkaline phosphatase signal was detected with BCIP/NBT reagents (Carl Roth, Milano, Italy, cat. 298-83-9 and 6578-06-9).

The immunoblotting detection was carried out using anti-AR (Cell Signaling, Pero, Italy, cat. D6F11), anti-AhR (Invitrogen, Monza, Italy, cat. MA1-514), anti-STAT3 (Cell signaling, Pero, Italy, cat. 124H6), anti-pY^705^STAT3 (Cell Signaling, Pero, Italy, cat. D3A7), anti-pS^139^H2AX (Santa Cruz Biotechnology, Segrate, Italy, cat. 101696), anti-H2AX (Santa Cruz Biotechnology, Segrate, Italy, sc-54606) anti-β-actin (Sigma-Aldrich, Milano, Italy, cat. A1978 clone AC-15), and anti-lamin A (Abcam, Cambridge, UK, cat. AB26300) primary antibodies diluted according to manufacturer’s instruction. Each experiment was replicated at least three times [9].

### 2.3. Immunofluorescence

Immunofluorescence analysis was performed essentially according to Cocchiola et al. [9]. Cultured cells were grown on coverslips and treated with β-HCH or testosterone following or not bicalutamide pretreatment upon the same aforementioned experimental conditions. Cells grown on coverslips were washed with PBS, fixed with 4% formaldehyde for 15 min, and then rinsed with PBS. Cells were permeabilized with cold methanol (−20 °C) for 5 min. After washing three times with PBS, the cells were blocked overnight with 3% *w*/*v* BSA (Sigma-Aldrich, Milano, Italy) in PBS. Fixed cells were processed for immunofluorescence staining to detect the localization of AR and AhR using anti-AR (Cell Signaling, Pero, Italy, cat. D6F11) and anti-AhR (Invitrogen, Monza, Italy, cat. MA1-514) as specific primary antibodies diluted in PBS containing 2% *w*/*v* BSA for 1 h. Following three washes with PBS added to with 0.05% Triton and 2% *w*/*v* BSA (PBS-T), cells were incubated for 1 h in the darkness with an FITC-conjugated secondary antibody (Jackson Immunoresearch, Milano, Italy, AlexaFluor 488-conjugated, cat. 211-545-109, dilution 1:800). Cell nuclei were counterstained with 100 ng/mL Hoechst (Sigma-Aldrich, Milano, Italy, cat. 94403) for 15 min. After washing with PBS-T, coverslips were mounted on glass microscope slides with Duolink^TM^ Mounting Medium and examined using a fluorescence microscope (Leica AF6000 Modular System, Milan, Italy) with 63× oil immersion objective. 

### 2.4. Extraction of RNA and RT-PCR

Total RNA was extracted from treated cells using TRIzol reagent (Invitrogen, cat. 15596026) in accordance with the manufacturer’s instructions as already described by Rubini et al. [7]. RNA was quantified spectrophotometrically, and its quality was assessed by 1.5% agarose gel electrophoresis and staining with ethidium bromide. The reverse transcription was carried out by Thermo Scientific RevertAid First Strand cDNA Synthesis Kit (Thermo Fisher Scientific, Monza, Italy, cat. K1622) in accordance with the manufacturer’s instructions. Gene expression was evaluated with specific primers for (cat. NM_001101 and NM_001648, Qiagen, Milano, Italy) using CFX ConnectTM Real-Time PCR Detection System (BioRad, Segrate, Italy) with a SYBR-Green fluorophore based real-time reaction (Brilliant SYBR Green QPCR Master Mix, Thermo Fisher Scientific, Monza, Italy). Gene expression analysis was performed using CFX ManagerTM Real Time PCR Detection System Software, Version 3.1 (BioRad).

### 2.5. Reactive Oxygen Species (ROS) Detection

Reactive oxygen species (ROS) generated by stressing cells with 75 µM tBOOH were quantified using the CellROX Green Flow Cytometry Assay Kit (Thermo Fisher Scientific, Monza, Italy, cat. C10492) following the manufacturer’s instructions. Samples were analyzed by a BD Accuri C6 flow cytometer (BD Biosciences, Milano, Italy).

### 2.6. Statistical Analysis

The repeatability of results was confirmed by performing all experiments at least three times. The obtained values are presented as mean and standard deviation. Statistical analysis was performed with GraphPad Prisma software using a Student’s *t*-test.

### 2.7. Determination GSH/GSSG

Reduced (GSH) and oxidized (GSSG) glutathione were measured by HPLC-UV according to Marrocco et al. [10]. Briefly, LNCaP and HepG2 cell pellets (1 × 10^6^ cells) were suspended in 10% ice-cold TCA and centrifuged for 15 min at 9000× *g*. The supernatant was collected and GSH and GSSG were measured by HPLC using a poroshell 120 EC-C18 column (3 × 150 mm, 2.7 µm) with UV detection at 215 nm. The mobile phase consisted of two solvent systems (A: 0.1% trifluoroacetic acid in water, and B: 100% 0.1% trifluoroacetic acid in water/acetonitrile 93:7) and the separation was achieved at a flow rate of 0.8 mL/min with the following elution gradient: 0–3 min 100% A + 0% B, 3–10 min from 100% A to 100% B.

### 2.8. Determination of the Lactate/Pyruvate Ratio

Determination of the lactate/pyruvate ratio was performed essentially according to Marrocco et al. [10] Lactic acid and pyruvic acid were analyzed by GC–MS as methoxime/tertbutyldimethylsilyl derivatives as previously described by Paik et al. [11]. GC-MS analyses were performed with an Agilent 6850A gas chromatograph coupled to a 5973N quadrupole mass selective detector (Agilent Technologies, Palo Alto, CA, USA). Chromatographic separations were carried out with an Agilent HP5ms fused-silica capillary column (30 m × 0.25 mm i.d.) coated with 5%-phenyl/95%-dimethylpolysiloxane (film thickness 0.25 µm) as stationary phase, using helium as the carrier gas at a constant flow rate of 1.0 mL/min, splitless injection mode at a temperature of 280 °C, and the following column temperature program: 70 °C (1 min) then to 300 °C at a rate of 20 °C/min and held for 10 min. The spectra were obtained in the electron impact mode at 70 eV ionization energy (ion source 280 °C and ion source vacuum 10−5 Torr). MS analysis was performed simultaneously in TIC (mass range scan from *m*/*z* 50 to 600 at a rate of 0.42 scans s^−1^) and SIM mode. GC-SIM-MS analysis was performed selecting the following ions: *m*/*z* 174 for pyruvate, *m*/*z* 261 for lactate, and *m*/*z* 239 for 3,4-dimethoxybenzoic acid (internal standard). Results were normalized on cell number and expressed as fold change relative to control samples.

## 3. Results

The cell lines used in this study (HepG2 and LNCaP) have suitable characteristics for testing the toxicological potential of β-HCH in all its facets, as well as being the same lines already employed as a model to demonstrate the hub role of STAT3 in the signaling pathways triggered by β-HCH. In addition, it should be remarked that the chosen experimental concentration of 10 µM for β-HCH derives from an epidemiological study that is biomonitoring the plasma concentration of β-HCH among people living in the industrial area of “Valle del Sacco”, south of Rome [7].

### 3.1. β-HCH as an Endocrine-Disrupting Chemical

On the basis of their physical characteristics and chemical structure, many pesticides can mimic or block the transcriptional activation elicited by naturally circulating hormones through the binding to hormone receptors, thus inducing an imbalance in intracellular homeostasis with consequent alterations in the endocrine system functions [12]. These molecules have been identified as endocrine-disrupting chemicals (EDCs).

Data regarding a possible role of β-HCH as an EDC are scarce and controversial, therefore shedding light on this mechanism could provide a further element to draw the toxicological profile of this substance. To understand whether β-HCH can interact with the androgen receptor (AR) signaling in the guise of agonist or antagonist, an effective experimental approach consists in following AR nuclear translocation by immunoblot and immunofluorescence upon treatment of LNCaP cells with 10 µM β-HCH.

**Figure 1:**
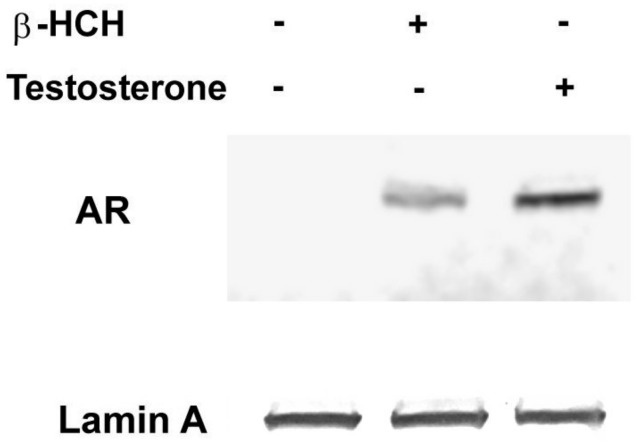
Androgen receptor (AR) nuclear localization in LNCaP cells. Western blot analysis was performed on nuclear extracts obtained from LNCaP cells exposed for 4 h to 10 µM β-hexachlorocyclohexane (β-HCH) or 30 nM testosterone. Treated samples exhibit an increase in AR nuclear localization compared to the control. Lamin A was used as a specific nuclear marker and normalization protein. The experiment was repeated three times and a representative blot is reported.

In this respect, LNCaP cells were incubated for 4 h with β-HCH or testosterone as a positive control; then nuclei were purified, and the extracts were analyzed by immunoblotting. As is clear from Figure 1, AR is detectable in the nuclear fraction of both β-HCH and testosterone treated samples, giving reason to believe that β-HCH can act as an AR agonist.

**Figure 2:**
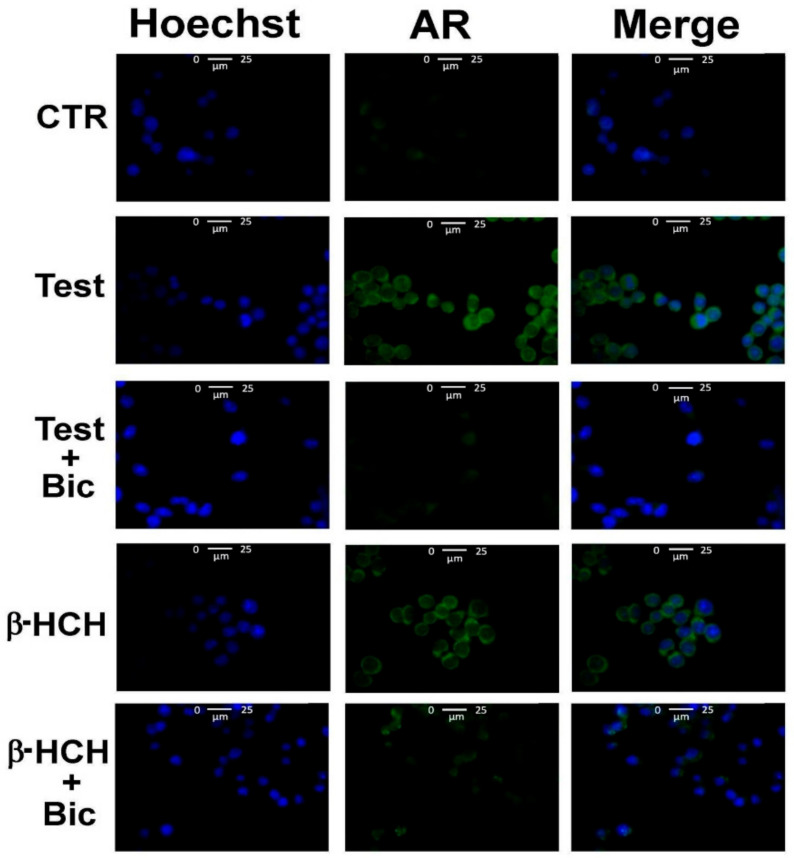
Cellular distribution of AR followed by immunofluorescence in LNCaP cells. CTR: Control untreated cells; Test: Cells subjected to a 4-h stimulation with 30 nM testosterone; β-HCH: Cells subjected to a 4 h stimulation with 10 µM β-HCH; Bic: Cells treated overnight with 120 nM bicalutamide. Samples referring to the images in the third and fifth rows of the panel (“Test + Bic” and “β-HCH + Bic”) were pretreated overnight with 120 nM bicalutamide and then subjected to a 4 h stimulation with β-HCH or testosterone AR nuclear localization induced by both β-HCH and testosterone, as evidenced by the images in the second and fourth rows, results inhibited by bicalutamide pretreatment.

**Figure 3:**
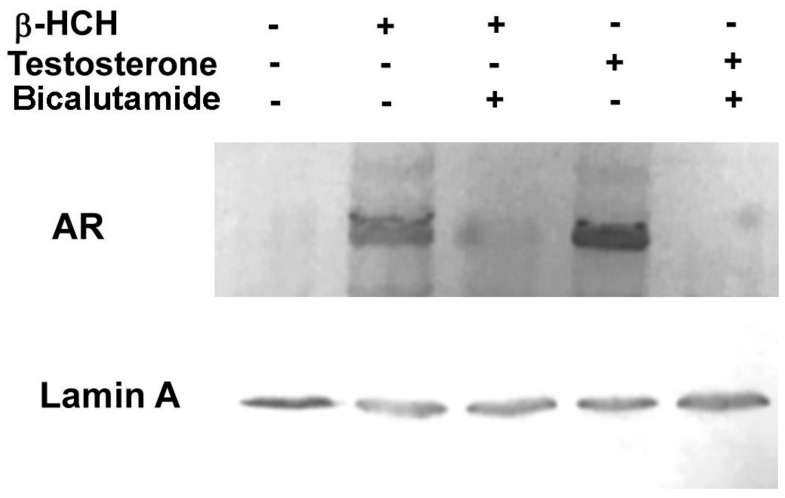
AR nuclear localization detected by Western blot analysis. Nuclear protein extracts were obtained from LNCaP stimulated for 4 h with 10 µM β-HCH or 30 nM testosterone, either pretreated or not with 120 nM bicalutamide incubation overnight. AR is found localized in the cellular nuclear fraction of samples treated with β-HCH and testosterone, whereas the band is barely detectable when cells are preincubated in the presence of the AR inhibitor bicalutamide. Lamin A was used as a specific nuclear marker and normalization protein. The experiment was repeated three times and a representative blot is reported.

According to these evidences, LNCaP cells were subjected to immunofluorescence analysis and AR was found to localize in the nucleus upon treatment with β-HCH or testosterone (Figure 2). In further confirmation of this hypothesis, the same experiment was repeated with an additional pretreatment step using the chemotherapeutic agent bicalutamide, an AR competitive inhibitor approved for prostate cancer therapy under the trade name Casodex [13]. The relative Western blot (Figure 3) confirms the capability of bicalutamide to block AR nuclear translocation induced by testosterone and β-HCH, providing extra evidence of β-HCH endocrine-disrupting potential.

**Figure 4:**
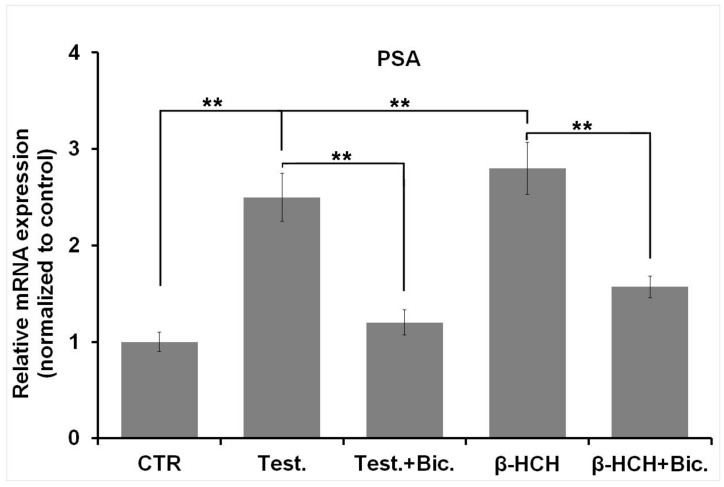
mRNA expression levels for PSA (prostate specific antigen) were analyzed by RT-qPCR. The exposure of LNCaP cells for 4 h to 10 µM β-HCH or 30 nM testosterone results in a two-fold PSA overexpression compared to the control untreated cells. Overnight pretreatment with 120 nM bicalutamide largely prevents the increase in PSA mRNA level. PSA expression values were normalized to β-actin as a housekeeping gene and expression levels of untreated cells were set to 1. CTR: Control untreated cells; Test: Cells subjected to a 4 h stimulation with 30 nM testosterone; β-HCH: Cells subjected to a 4 h stimulation with 10 µM β-HCH; Bic: Cells treated overnight with 120 nM bicalutamide. Statistically significant differences (** *p* < 0.01) are marked with asterisks.

To check if β-HCH could also activate AR from a transcriptional point of view and affect the expression of AR-target genes, the mRNA level of PSA (prostate specific antigen) was evaluated through RT-qPCR. Apart from being a biomarker of choice for prostate cancer, PSA is an AR-dependent gene and therefore constitutes a good candidate to verify AR activity as a transcription factor [14,15]. RT-qPCR analysis was then carried out to measure PSA mRNA levels: As displayed in Figure 4, PSA gene is overexpressed in samples treated with β-HCH and testosterone, but its expression is decreased in the presence of bicalutamide. These evidences strongly support the AR agonism of β-HCH.

### 3.2. Activation of the AhR Pathway

AhR is a cell nuclear receptor that acts as a ligand-activated transcription factor involved in the recognition and detoxification of a wide range of non-physiological and structural divergent chemicals commonly referred to as “xenobiotics” [16]. Most of the research particularly focuses on AhR function as a mediator of biochemical responses to organochlorine compounds, first and foremost dioxin, for deepening the impact of AhR activation on cellular adaptive mechanisms [17]. Upon agonist binding, AhR translocates to the nucleus where it binds DNA-responsive elements to control the expression of genes coding for metabolizing enzymes (e.g., CYP450) needed for the clearance of foreign substances from the body [18,19]. After exerting its biological effects, AhR is ubiquitinated and subjected to proteasome-mediated degradation [20].

**Figure 5:**
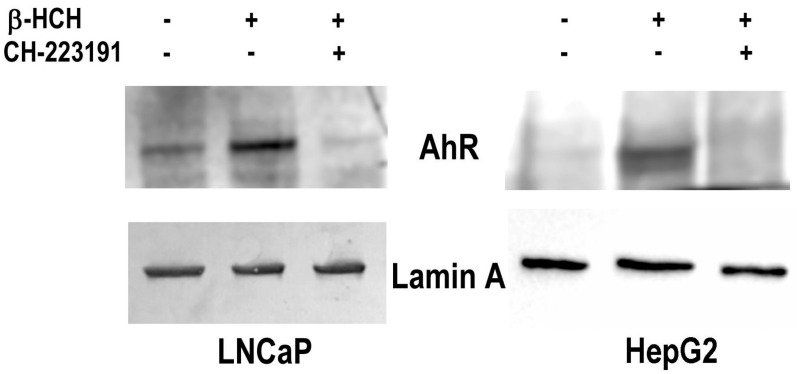
AhR nuclear localization in LNCaP and HepG2 cells. Western blot analysis was performed on nuclear protein extracts obtained from both prostate (LNCaP) and hepatic (HepG2) cancer cellular models treated with 10 µM β-HCH following or not a 2 h pretreatment with 150 nM CH223191. The presence of AhR in the nuclear fraction is more noticeable in the samples corresponding to β-HCH stimulated cells with respect to control untreated cells. The amount of nuclear AhR is similar to control if cells are treated with 10 µM β-HCH in the presence of the AhR antagonist CH223191. Lamin A was used as a specific nuclear marker and normalization protein. The experiment was repeated three times and a representative blot is reported.

**Figure 6:**
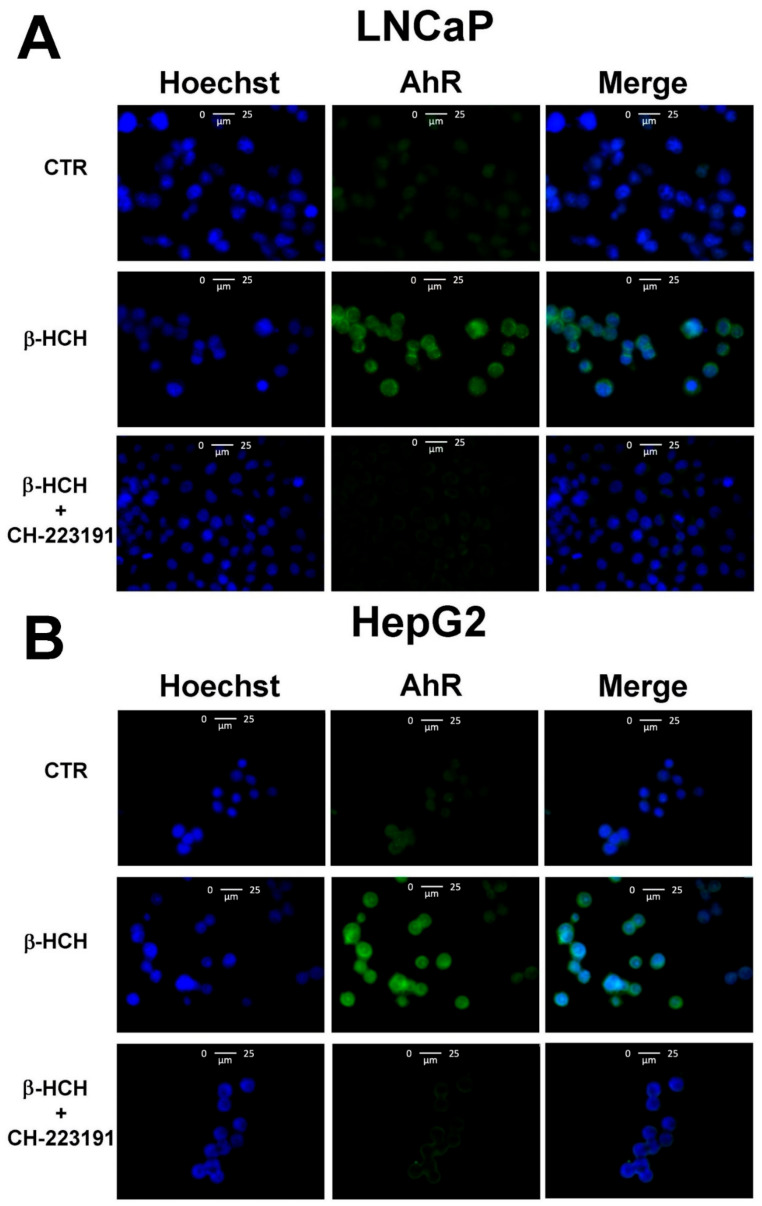
Cellular distribution of AhR followed by immunofluorescence in LNCaP (**A**) and HepG2 (**B**) cells. CTR: Control untreated cells; β-HCH: Cells after 4 h of 10 µM β-HCH stimulation; β-HCH + CH223191: Cells after 2 h pre-incubation with 150 nM CH223191 followed by 4 h of 10 µM β-HCH stimulation.

To demonstrate the β-HCH-dependent activation of AhR genomic pathway, AhR nuclear localization was verified through immunoblotting performed on nuclear extracts obtained from both LNCaP and HepG2 cells exposed to β-HCH and pre-treated or not with the AhR antagonist CH223191 [21]. As reported in Figure 5, the receptor is only present in the nuclear fractions of β-HCH treated samples whereas no bands are detectable in the nuclear fractions relative to control and CH223191 pre-treated specimens. To support this finding, immunofluorescence analysis was performed under the same experimental conditions on both LNCaP and HepG2 (Figure 5; Figure 6).

**Figure 7:**
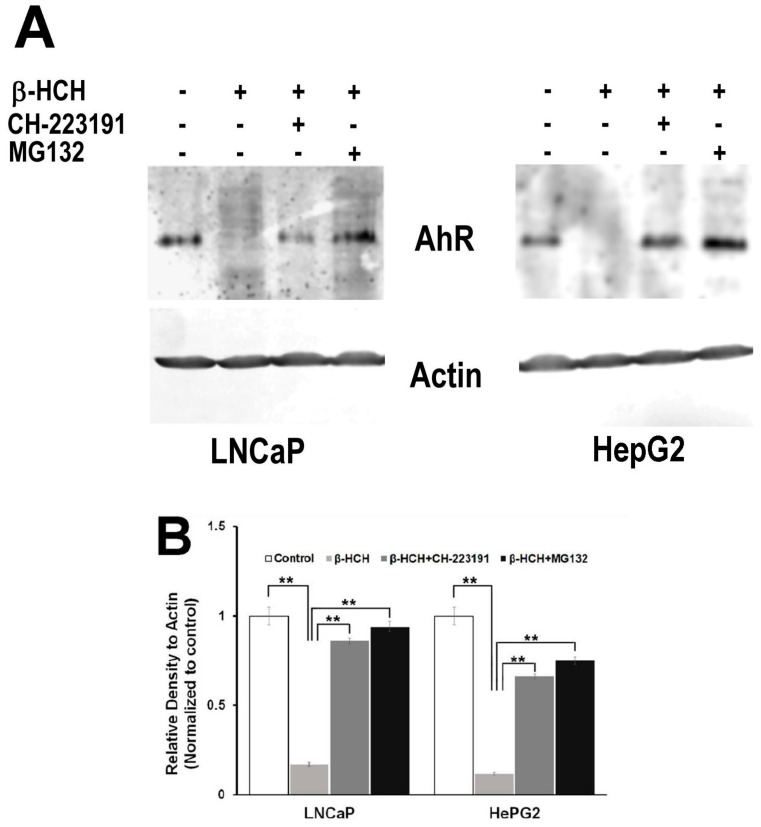
AhR proteasomal degradation is induced by β-HCH in both LNCaP and HepG2 cells. (**A**) Total protein extracts were subjected to Western blot analysis. Samples obtained from cells treated with 10 µM β-HCH alone exhibit a weaker signal compared to the samples from cells treated with both the inhibitors. The 2 h pretreatment step with 2 µM MG132 or 150 nM CH22131 is able to maintain AhR at the same levels as the control. (**B**) Quantification of protein levels by densitometric analysis. Values for each sample were normalized against the β-actin level present in the same sample and taken as a housekeeping control. The experiment was repeated three times and a representative blot is reported. Statistically significant differences (** *p* < 0.01) are marked with asterisks.

These outcomes constitute a first confirmation of AhR activation induced by β-HCH. In parallel, to establish if β-HCH can promote AhR degradation, either LNCaP and HepG2 cells were incubated with β-HCH following pre-treatment with the proteasome inhibitor MG-132 [22] or with CH223191, and total cellular protein extracts were subjected to immunoblotting. The lane loaded with lysates from β-HCH treated cells reveal an evident AhR degradation that is not observable in samples incubated together with MG-132 or CH223191 (Figure 7). These results suggest that β-HCH could directly interact with AhR, activating it and allowing its subsequent degradation via the ubiquitin–proteasome system.

### 3.3. Oxidative Stress and Energy Metabolism

Several studies correlate the toxicity of organochlorine compounds to their capability of inducing the formation of reactive oxygen species (ROS) responsible for the generation of oxidative stress [23].

Oxidative stress is a cellular condition caused by an imbalance between oxidants and antioxidants with a loss of ability of a biological system to maintain the redox homeostasis [24]. This has a strong impact on the entire organism as a consequence of structural and functional alterations of key biomolecules [25].

**Figure 8:**
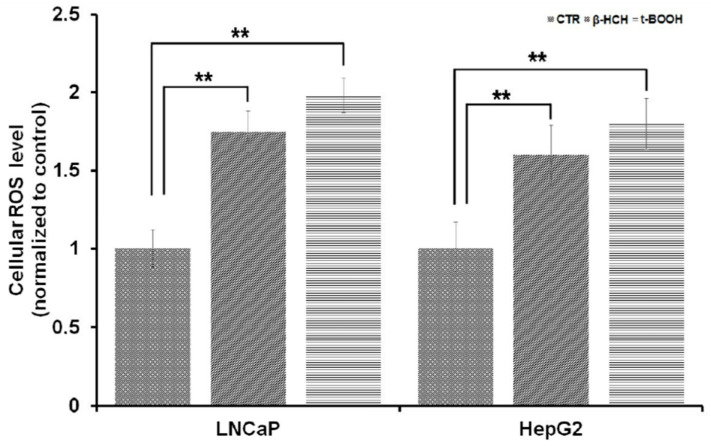
Reactive oxygen species (ROS) production detected by CellROX assay. The histogram shows an approximately two-fold increase in fluorescence intensity in samples treated with 10 µM β-HCH for 6 h compared to untreated cells. 75 µM Tert Butyl Hydro peroxide was used as a positive control for ROS induction. Statistically significant differences (** *p* < 0.01) are marked with asterisks.

A recently published article provided evidence that 20 µM β-HCH can induce a substantial ROS increase in HOSE ovary cells [26]. To confirm this outcome, ROS production was quantified by performing CellRox assay on both LNCaP and HepG2 cells treated with 10 µM β-HCH for 6 h. Values listed in Figure 8 evidence a significant intensification of the fluorescence after β-HCH stimulation, thus indicating an enhanced ROS production.

**Figure 9:**
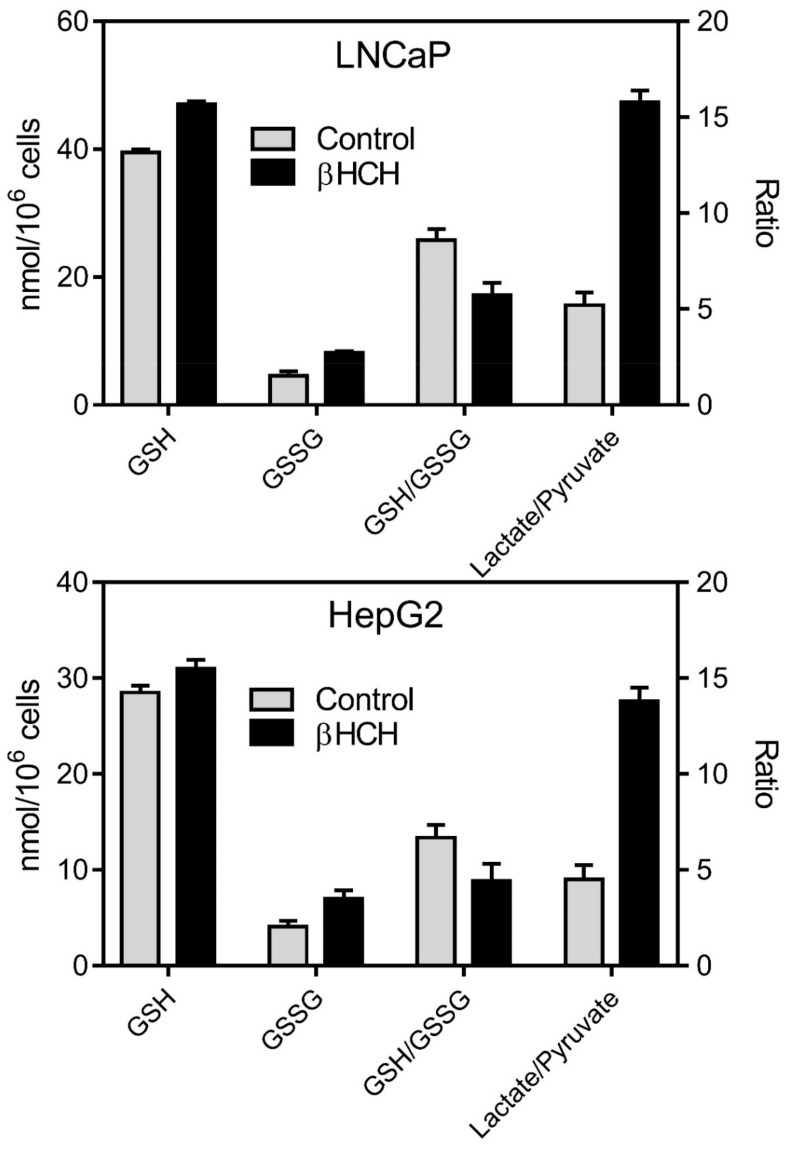
Impact of β-HCH on glutathione redox state and cell metabolism. The increase in glutathione oxidized form (GSSG), with a consequent decrease in GSH (reduced glutathione)/GSSG ratio, proves that 10 µM β-HCH can induce oxidative stress after 6 h treatment in both LNCaP and HepG2 cells. Analysis were carried out on 10^6^ cells. In addition, a sharp increase in Lactate/Pyruvate ratio is detectable in the culture media of both LNCaP and HepG2 cells stimulated with 10 µM β-HCH for 6 h, demonstrating an enhancement in lactate production induced by β-HCH. All the differences between control and β-HCH treated samples are statistically significant.

Taking into account that glutathione redox status constitutes another reliable biomarker of oxidative stress [27,28,29], the same samples were subjected to measurement of the GSH/GSSG ratio. Results reported in Figure 9, show a marked increase in the glutathione oxidized form (GSSG), with a consequent decrease in GSH/GSSG ratio, demonstrating the induction of oxidative stress in response to β-HCH. The establishment of an overall oxidative stress condition is often associated with a reprogramming of cellular bioenergetics [30]; for this reason, additional studies are needed to evaluate the extent to which an imbalance in redox homeostasis is reflected in energy metabolism.

Highly aggressive tumors are likely to display a particular metabolic condition known as aerobic glycolysis or Warburg Effect, characterized by the preferential conversion of pyruvate to lactate, rather than to acetyl-CoA, even in normoxia [22,31]. In this context, the potential impact of β-HCH on cell metabolism was inspected by determining the lactate/pyruvate ratio in the culture media of cells treated or not with β-HCH. As is clear from Figure 9, lactate is predominant in stimulated samples, attesting the influence of β-HCH molecular action on cellular metabolic rewiring.

### 3.4. γ-H2AX as an Indicator of β-HCH Induced Genotoxicity

**Figure 10:**
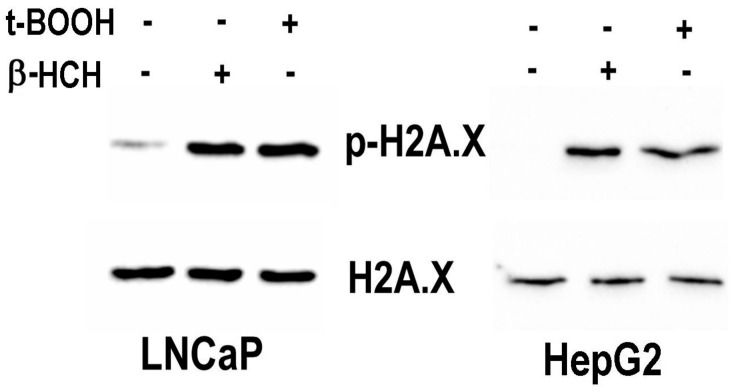
β-HCH induces the phosphorylation of H2AX at Serine 139. Nuclear protein extracts were obtained from both LNCaP and HepG2 exposed to 10 µM β-HCH for 4 h or 75 µM t-BOOH for 1 h used as a positive control for DNA damage. The immunoblot shows a comparable increase in the intensity of the band corresponding to phosphorylated H2AX in samples treated with β-HCH as well as t-BOOH in either LNCaP or HepG2 cells. Each sample was normalized against the unmodified H2AX levels present in the same protein extract. The experiment was repeated three times and a representative blot is reported.

The relationship between DNA damage and sustained exposure to environmental pollutants is widely described in the scientific literature and is probably linked to the redox signaling triggered by OCPs [32]. For some pesticides, the processes leading to alterations in the cellular homeostasis are partially understood, but commonly recognized mechanisms include their enzymatic conversion to secondary reactive products, depletion of cellular antioxidant defenses and/or impairment of antioxidant enzyme functions [33]. The phosphorylation of histone H2AX at serine 139 is a post-translational modification that constitutes a solid and versatile endpoint to investigate the genotoxic potential of a chemical [34]. LNCaP and HepG2 cells were stimulated with β-HCH or t-BOOH as positive control [35] and nuclear fractions subjected to immunoblotting. With respect to the untreated control, H2AX results phosphorylated following β-HCH exposition in both the considered cell lines (Figure 10).

## 4. Discussion

β-HCH is the environmental heritage of unscrupulous industrial policies: In fact, although technical-grade lindane production ceased in the late 1960s [36] and its use was definitively banned by the Stockholm Convention in 2009, β-HCH still remains one of the most widespread organochlorine pesticides accounting for about 7.2 tons illegally buried all over the world. The expression “small molecule with a big impact” sums up the wide range of pathologic effects of β-HCH on cellular processes (Figure 10). For decades β-HCH has been a perfect stranger among organochlorine compounds, but in recent years has caught the attention of both academia and civil society because of its suspected harmfulness to humans. Differently from the other HCH isomers, the spatial arrangement of the chlorine atoms on the cyclohexane ring confers to β-HCH stronger chemical stability and lipophilic properties which, in turn, contribute to the long biological half-life and high bioaccumulation tendency of this pollutant [3]. On the other hand, its small size and its low structural similarity with lipophilic hormones virtually make β-HCH an inefficient endocrine disruptor compared with best-known OCPs such as dioxin or DDT. It should however be considered that β-HCH levels detected in the blood of the exposed population are approximately 100 to 1000 times greater than the hormones physiological concentration, that is within the nanomolar range [37]. Hormones are secreted in response to precise physiological stimuli and their concentration fluctuates with the rhythms or biological needs; for example, after a physical exercise an increase in the concentration of cortisol is observed [38]. Instead, β-HCH is constantly present in the organism with a concentration clearly higher compared to that of physiological hormones: This allows its interaction with the hormonal receptors more due to a mass action than to its specificity for the target.

β-HCH at a final concentration value of 10 µM, extrapolated from the aforementioned epidemiological investigation [8], has proven to be able to induce AR nuclear translocation and transcriptional activation in AR^+^ prostate cancer cells (LNCaP). In addition, PSA gene, which is a specific marker for prostate cancer under AR transcriptional control, results similarly upregulated upon β-HCH and testosterone treatment, whereas its mRNA levels decrease when cells are pre-treated with the AR antagonist bicalutamide. Taken together, these evidences strongly support the endocrine disrupting properties of β-HCH.

Since it is also a full-on xenobiotic, the predictable β-HCH-dependent activation of the AhR signaling was investigated by performing experiments on both LNCaP and HepG2 cells. Outcomes indicate that β-HCH promotes AhR nuclear localization as well as its subsequent degradation via ubiquitin–proteasome pathway. To confirm this hypothesis, the AhR antagonist CH223191 and the proteasome inhibitor MG-132 were used as a pre-treatment step before β-HCH stimulation, revealing that AhR degradation occurs in the presence of β-HCH but not with CH223191 or MG-132; furthermore, CH223191 counteracts the increase in AhR expression levels induced by β-HCH.

As already demonstrated in a previous study from our laboratory, β-HCH can activate multiple cell line-specific pathways converging toward a higher proliferation rate, thus contributing to the development of oxidative stress [7]. The establishment of this cellular condition is testified by an increase in ROS levels and an imbalance in the GSSG/GSH ratio in favor of the glutathione oxidized form with consequences on cellular bioenergetic profile, and promotes the metabolic shift to aerobic glycolysis, resulting in an enhanced production of lactate typically associated with more aggressive tumor types. In this altered context, the histone H2AX is phosphorylated at Serine 139 (γH2AX) evidencing the occurrence of a β-HCH-induced DNA damage.

Taking into consideration that scientific literature lacks molecular and cellular studies on β-HCH, these experimental results provide significant elements to further characterize the biochemical behavior of this pollutant and its impact on human cellular processes.

## 5. Conclusions

In the employed cellular models (LNCaP and HepG2), β-HCH can simultaneously activate multiple cellular processes such as hormonal and AhR pathways, oxidative stress, energy metabolism and DNA damage. All these processes are responsible for the transformation of the tumor phenotype into a more undifferentiated and more aggressive form.

**Figure 11:**
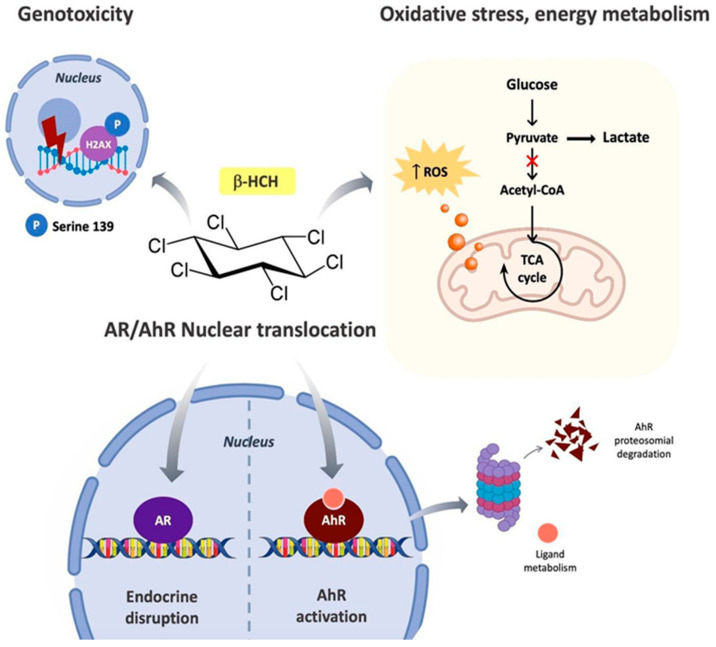
The multifaceted effects of β-HCH on intracellular biochemical processes.

In fact, due to its marked lipophilia, β-HCH is a bioaccumulating xenobiotic that, following environmental exposure, maintains constant levels of concentration significantly higher with respect to its functional-related biomolecules (i.e., hormones, growth factors), which are produced depending on the physiological needs of the organism. These characteristics allow β-HCH to exert its activities at different molecular levels, ranging from signal transduction (STAT3 activation), as described in our previous article [7], to redox homeostasis (ROS and GSSG/GSH ratio), energy metabolism (lactate/pyruvate ratio), and endocrine system (AhR/AR axis), as schematized in Figure 11. Considering all these scientific evidences, β-HCH represents a ticking biological time-bomb waiting to be defused. For this purpose, removal and bio-degradative strategies should be investigated pointing towards the intrinsic and environmental resilience of β-HCH as major burden. Since several HCH-degrading systems have already been identified in particular for the γ-isomer [39], in the future they may also be applied to β-HCH.
